# Assessment of genetic familiarity and genetic knowledge among Palestinian university students

**DOI:** 10.1186/s12909-023-04996-6

**Published:** 2024-01-03

**Authors:** Maha Rabayaa, Mustafa Ghanim, Yasmeen Saleh, Mohammad Abuawad, Ramzi Shawahna

**Affiliations:** 1 Department of Biomedical Sciences, Faculty of Medicine and Health Sciences, An-Najah National University, PO. Box 7, Nablus, West Bank Palestine; 2 School of Pharmacy, University of Southern California, Los Angelos, CA USA

**Keywords:** Consanguinity, Familiarity, Genetics, Knowledge

## Abstract

**Background and aims:**

Genetic knowledge and familiarity among the population represent the lane toward effective participation in social decisions regarding genetic issues. This cross sectional research aimed to assess genetic knowledge and familiarity among university students in Palestine.

**Methods:**

The familiarity with genetics was evaluated using the Genetic Literacy and Comprehension instrument (GLAC), and genetic knowledge was measured using a 16-item scale of prevalent genetic concepts.

**Results:**

Among the 624 participants, 59.5% were females. 38.8% reported family history of genetic diseases. The genetic familiarity mean score was 4.83 and the genetic knowledge mean total score was 11.5. Students’ genetic familiarity was high for the terms chromosome and genetic while it was low for the terms sporadic and vulnerability. Genetic knowledge was highest for gene definition while it was the lowest regarding the number of human genes. The age group, year of study, and learning genetic courses were the significant predictors of familiarity among medical students. The year of study, family history of genetic diseases, parental consanguinity, and learning genetic courses were the significant variables associated with genetic knowledge among medical students. Regarding the non-medical group of participants, all study variables were significant for both familiarity and knowledge scores except for age group with familiarity.

**Conclusion:**

Genetic familiarity and knowledge among Palestinian university students are inadequate. Consanguinity and hereditary disorders are prevalent in Palestine. These findings encourage university stakeholders to take action to improve genetic knowledge and familiarity among students through both appropriate pedagogical and non-pedagogical interventions.

## Introduction

Several genetic disorders show an increased incidence rate rate in the Arab world including Palestine [1]. This could be attributed to multiple social factors related to marriage and fertility issues. In particular, consanguinity is still widely practiced and might reach 50% of the total marriages in the Palestinian population according to some studies [1, 2]. In addition, the tendency of Palestinians to have an extended family led to increased maternal and paternal ages at conception, which is consequently associated with serious genetic disorders [3].

Genetic disorders, due to their severity and the inability to treat them, impose major health, economic, and social challenges on individuals and communities. Therefore, genetic knowledge among Palestinians is a top priority issue. Nevertheless, there is insufficient progress in this field in Palestine while genetic counseling and education are getting greater attention globally [4, 5].

Moreover, there is a lack of unified preventive programs at the national level for prenatal care, screening, diagnosis, and management of genetic anomalies [6]. Furthermore, previous studies have shown that there is insufficient public knowledge about genetic disorders, and families with affected members consistent with genetic disorders were shown to be stigmatized [3]. Thus, evaluating public genetic knowledge and familiarity is a crucial step. Delivering genetic counseling services to the general population in Palestine poses a significant challenge due to cultural factors that can adversely affect counseling outcomes. These factors include the central role of the family in Arab culture, where marriage is considered a family affair. Additionally, gender differences in decision-making and the importance of religion as an integral component of Arab culture can further complicate the process of delivering effective genetic counseling [7].

The accelerated recognition of the role of genetics in medicine became evident with the discovery of not only rare genetic diseases but also common chronic conditions such as cancer, cardiovascular diseases, and metabolic disorders [8]. Recently, significant advancements have taken place in the fields of genetic analyses, technology to generate and spread information about genetic disorders, and genetic counseling [9]. As a direct consequence of these advances, delivering the decisions of health care professionals to patients with genetic disorders is influenced by the patient’s knowledge regarding medical genetics [10]. Incorporating genetic knowledge of basic human genetic principles and medical genetics aids in understanding genetic disorders and achieving proper health-related decisions [11]. Measuring genetic familiarity with genetic terminology, clinical skills, and factual knowledge about genes and hereditary traits is important [12]. Evidently, a basic amount of genetic knowledge is essential to understanding and interpreting the results of genetic analyses. Several studies focused on assessing the impact of genetic knowledge on the population’s perception of genetic disorders [13, 14].

In Palestine, there is a previous study that investigated the level of genetic knowledge among parents of children with genetic disorders [3]. To the best of our knowledge, the current study is the first one to assess genetic familiarity and knowledge among Palestinian university students. University students were recruited from a variety of disciplines because it was anticipated that they have varying educational backgrounds and represent a wide range of the Palestinian community. Indeed, the percentage of youth (18–29 years) in Palestine was found to be 23% in the West Bank of Palestine based on the Palestinian Central Bureau Statistics. More importantly, Palestinian university students are at a pre-marriageable age where critical decisions and proper planning should be made. Assessing the level of genetic knowledge and familiarity among university students has practical implications for healthcare and societal issues. In addition, this area of research contributes to the improvement of academic programs and policies related to genetics education in Palestine.

## Methods

### Study design and sampling

A cross-sectional questionnaire-based study was conducted from November to December 2022 to evaluate genetic familiarity and knowledge among Palestinian university students. Taking into consideration the total number of Palestinian university students in the West Bank is approximately one hundred and thirty-nine thousand students. The sample size was calculated at a 95% confidence interval (CI) and accepted a default margin of error of 5%. The minimal sample size was calculated as 383 participants using an online sample size calculator (www.raosoft.com). A convenient sample of students from ten Palestinian universities in the West Bank which have together a total number of around 120 thousand students (An-Najah National University, Arab American University, Birzeit University, Al-Quds University, Hebron University, Bethlehem University, Palestine Polytechnic University, Palestine Technical University-Kadoorie, Al-Quds Open University, and Palestine Ahliya University) was enrolled in the study after signing an informed consent form. These universities are distributed all over the area of the West Bank in which there are around 3.2 million habitants. The questionnaire was designed using Google Forms and distributed electronically via student sites on social media, student emails, and universities’ e-teaching tools. The questionnaire was distributed to student sites but not to the public platforms.

### Ethical consideration

The study was approved by the Institutional Review Boards of An-Najah National University (Ref. Med. Octo. 2022/42). Participation in this study was completely voluntary. An informed consent form was obtained from all participants. The informed consent form explained the premise of the study and assured the anonymity of the participants.

### Data collection tools

The questionnaire was structured based on previously published studies [12, 15]. The questionnaire contained three sections. The first section included the socio-demographic characteristics of the participants like age, gender, study field, study year, family history of genetic disease, parents’ consanguinity, attending genetic courses, and place of residence. The second section evaluated genetic familiarity using the GLAC instrument [16]. GLAC was designed for this study to assess familiarity with eight common genetic terms and concepts that are used in the short version of the Rapid Estimate of Adult Literacy in Genetics (REAL-G). However, REAL-G is used in clinical settings and aurally administered. Therefore, the GLAC was more suitable for this study.

The third section measured the actual genetic knowledge using 16 items about facts related to the association between genes, chromosomes, cells, the body, and diseases [17]. The instrument contains questions on basic genetic knowledge and postulates about the association between genes, chromosomes, and cells and the body and diseases which are in line with the ongoing advances in genetic knowledge. Furthermore, specialists in genetics were consulted to review the content of the questionnaire.

The questionnaire was reviewed and translated into the Arabic language by academicians who specialized in the field of genetics. A pilot study was carried out on 38 students to evaluate the questionnaire’s feasibility, timing, and reliability. The questionnaire was then further modified and distributed in both the translated Arabic version alongside the original English version, to make sure that each participant can articulate and respond appropriately to the questions.

### Genetic familiarity assessment

The genetic familiarity was evaluated using the GLAC instrument which assessed the familiarity of the participants with eight commonly used genetic terms: genetic, chromosome, susceptibility, mutation, variation, abnormality, heredity, and sporadic. The participants were asked to subjectively rate their general familiarity with each term on a Likert scale of 7 points (‘not familiar at all’ (1), ‘moderately unfamiliar’ (2), ‘slightly unfamiliar’ (3), ‘familiar’ (4), ‘slightly familiar’ (5), ‘moderately familiar’ (6), and ‘completely familiar’ (7)). Scores were constructed based on respondents’ average perceived familiarity across the eight items (Range: 1 to 8). The calculated Cronbach’s alpha value for the internal consistency of the familiarity scale was 0.90. A 70% cut-off value was used to indicate low/high familiarity based on previous reports and it was adopted according to Mokken scaling procedures to attain proper variability. Those who achieved a score of 70% or more (Range 5–7) were considered to have a high familiarity score [12, 16].

### Genetic knowledge assessment

Knowledge of genes and heredity was measured using 16 items. Participants answered either “Yes” when they believed the statement was true, “No” when they believed the statement was false, or “I do not know.” The answers to seven items out of sixteen were pre-designed to be incorrect. Each correct response received one point and none for either incorrect or I do not know responses. The total score ranged from 0 to 16. The Cronbach’s alpha value for the internal consistency of the genetic knowledge scale was 0.86. A 70% cut-off value was used to indicate low/high knowledge based on previous reports and it was adopted according to Mokken scaling procedures to attain proper variability. Those who achieved a score of 70% or more (Range 12–16) were considered to have a high knowledge score [12].

### Statistical analysis

Statistical analyses were performed using Statistical Package for the Social Sciences Statistics (SPSS) for Windows, version 21 (IBM Corp., Armonk, N.Y., USA). Descriptive analyses were applied for sociodemographic characteristics. The one-way ANOVA and t-test were conducted as appropriate to compare the effect of sample characteristics on the genetic familiarity and genetic values among all studied samples, including students in the medical field and non-medical fields. To control potential confounding factors, the variables that were significantly associated with the t-tests or the ANOVA were included in multiple linear regression models. Goodness-of-fit was evaluated using a significant R^2^. Variance inflation factor and tolerance were used to diagnose multicollinearity problems. A *p*-value of < 0.05 was considered statistically significant.

## Results

### Participants’ sociodemographic characteristics

This study included a total of 624 students from ten Palestinian universities. The average age of the respondents was 20.4 years old (SD ± 2.34). Females represented 59.5% of the sample. The specialties of the enrolled students included: human medicine-basic phase (19.2%), human medicine-clinical phase (14.1%), health sciences (33.5%), scientific nonmedical specialties (16.3%), and literature majors (16.8%). Approximately 38.8% of participants reported the presence of genetic diseases within their families. 36.1% reported the presence of parental consanguinity, and 48.2% reported having studied genetic courses at the university. Participants’ sociodemographic characteristics are presented in Table 1.

**Table 1:** Sociodemographic characteristics of the participants (*n* = 624)

Variable		Frequency (%)
Age groups	Less than 21 years	367 (58.8)
	21 years or more	257 (41.2)
Gender	Male	253 (40.5)
	Female	371 (59.5)
Year of Study	First	153 (24.5)
	Second	172 (27.6)
	Third	84 (13.5)
	Fourth	116 (18.6)
	Fifth	57 (9.1)
	Sixth	42 (6.7)
Field of study	Medicine, Basic phase	120 (19.2)
	Medicine, clinical phase	88 (14.1)
	Health Sciences	209 (33.5)
	Scientific, nonmedical specialties	102 (16.3)
	Literature majors	105 (16.8)
Family history of genetic disease	No	382 (61.2)
	Yes	242 (38.8)
Parents consanguinity	No	399 (63.9)
	Yes, the first cousin	128 (20.5)
	Yes, double-first cousin	44 (7.1)
	Yes, more distant relationship	53 (8.5)
Learning of genetic courses	No	323 (51.8)
	Yes	301 (48.2)
Place of residence	City	256 (41)
	Village	303 (48.6)
	Camp	65 (10.4)

The overall familiarity with genetic terms among participants was relatively low. The term chromosome was the most familiar while the term vulnerability was the least familiar term. The familiarity with other terms was variable and generally low. 61.1% and 54.2% of the participants were completely familiar with the terms chromosome and genetics, respectively. In contrast, only 12.2% and 17.8% of the participants showed complete familiarity with the terms vulnerability and sporadic, respectively. More than a quarter of the participants were entirely unfamiliar with the terms vulnerability and sporadic. Around 11.9%, 13.9%, 9.3%, 5.3%, 2.9%, and 2.7% were moderately familiar, slightly familiar, familiar, slightly unfamiliar, moderately unfamiliar, and not familiar at all with genetic terms respectively. Nearly 13.3%, 7.5%, 8.7%, 4.6%, 2.6%, and 2.2% of the participants reported being familiar with chromosome terms sequentially from being moderately familiar (6) to unfamiliar at all (1). 26.8% were not familiar at all with the term vulnerability. 43.1%, 37.7%, 42.1%, and 35.9% were completely familiar with the terms mutation, variation, abnormality, and hereditary, respectively. Results are shown in Table 2.

**Table 2:** Genetic familiarity among the participants

Familiarity terms	The subjective score of familiarity in genetics terms n (%)						
	1 ‘’Not familiar at all’’	2	3	4	5	6	7 ‘’Completely familiar’’
1. Genetic	17 (2.7)	18 (2.9)	33 (5.3)	58 (9.3)	86 (13.8)	74 (11.9)	338 (54.2)
2. Chromosome	14 (2.2)	16 (2.6)	29 (4.6)	54 (8.7)	47 (7.5)	83 (13.3)	381 (61.1)
3. Vulnerability	167 (26.8)	72 (11.5)	86 (13.8)	93 (14.9)	75 (12)	55 (8.8)	76 (12.2)
4. Mutation	59 (9.5)	45 (7.2)	52 (8.3)	66 (10.6)	56 (9)	77 (12.3)	269 (43.1)
5. Variation	76 (12.2)	52 (8.3)	52 (8.3)	67 (10.7)	63 (10.1)	79 (12.7)	235 (37.7)
6. Abnormality	68 (10.9)	55 (8.8)	44 (7.1)	64 (10.3)	60 (9.6)	70 (11.2)	263 (42.1)
7. Hereditary	74 (11.9)	46 (7.4)	70 (11.2)	63 (10.1)	60 (9.6)	87 (13.9)	224 (35.9)
8. Sporadic	185 (29.6)	74 (11.9)	64 (10.3)	78 (12.5)	65 (10.4)	47 (7.5)	111 (17.8)

Table 3 displays the results for the sixteen questions used to assess genetic knowledge. Among 624 participants, seven questions (1, 3, 4, 7, 8, 10, and 11) out of sixteen were correctly answered by more than 80% of the participants. More than 70% of participants correctly answered questions about genetic diseases (questions number 2, 5, 6, and 9) in addition to question 12 “A gene is part of a chromosome”. 63.9% of the participants know that chromosome is bigger than gene and 53.4% of the participants comprehend that the genome is susceptible to human intervention. The question about the variable expression of genes in different body parts was correctly answered only by 38.3% of the participants (question number 13).

**Table 3:** Genetic knowledge among university students

Genetic knowledge questions	Correct answer n (%)	Incorrect answer n (%)	I do not know n (%)
**1. One can see genes with the naked eye**	537 (86.1)	16 (2.6)	71 (11.4)
2. Healthy parents can have a child with a genetic disease	484 (77.6)	87 (13.9)	53 (8.5)
3. The onset of certain diseases is due to genes, environment, and lifestyle	526 (84.3)	29 (4.6)	69 (11.1)
**4. A gene is a disease**	528 (84.6)	38 (6.1)	58 (9.3)
5. The carrier of a disease gene may be completely healthy	470 (75.3)	67 (10.7)	87 (13.9)
**6. All serious diseases are hereditary**	492 (78.8)	73 (11.7)	59 (9.5)
7. A gene is a molecule that controls hereditary characteristics	540 (86.5)	26 (4.2)	58 (9.3)
8. Genes are inside cells	503 (80.6)	51 (8.2)	70 (11.2)
**9. The child of a disease gene carrier is always also a carrier of the same disease gene**	439 (70.4)	89 (14.3)	96 (15.4)
10. A gene is a piece of DNA	532 (85.3)	41 (6.6)	51 (8.2)
**11. A gene is a cell**	511 (81.9)	43 (6.9)	70 (11.2)
12. A gene is part of a chromosome	449 (72)	73 (11.7)	102 (16.3)
13. Different body parts include different genes	239 (38.3)	222 (35.6)	163 (26.1)
**14. Genes are bigger than chromosomes**	399 (63.9)	82 (13.1)	143 (22.9)
**15. The genome is not susceptible to human intervention**	333 (53.4)	112 (17.9)	179 (28.7)
16. It has been estimated that a person has about 20,000–22,000 genes	188 (30.1)	120 (19.2)	316 (50.6)

*The correct answer for the questions in bold is false

### Genetic familiarity and knowledge variation based on sample characteristics among all participants

The mean familiarity score of the eight genetic terms was 4.83 (SD = 1.5) while the median was 4.88. The mean total knowledge score of the 16 items was 11.5 (SD = 3.6) while the median was 13. Using a 70% cut-off value, 49.4% of the students achieved high familiarity scores (the mean score of five or more) and 64.3% achieved high total scores in knowledge (total score of 12 to 16). The familiarity scores were significantly variable based on all studied variables (p-value < 0.05). The mean was higher in students aged 21 years or older compared with younger students. Females achieved more familiarity than males. Students in their sixth year of study showed significantly the highest mean compared with students in other years of study. Students who were in the clinical phase of the human medicine specialty achieved the highest mean when compared with other specialties. Furthermore, higher mean ranks were reported in students who did not have a family history of genetic diseases compared with those who did. The students whose parents did not have consanguineous relativity showed a significantly higher familiarity with genetics than those who reported any degree of parental consanguinity. Students who had learned genetic courses had significantly higher familiarity with genetic terms. Finally, those who were residents of cities reported more genetic familiarity. Genetic knowledge scores were significantly variable based on all studied variables (p-value < 0.05). The mean of the total genetic knowledge score was higher in students whose ages were less than 21 years old in comparison to older students. Students in the second year of study achieved a higher total score of genetic knowledge. Those who were studying the basic phase of human medicine achieved higher scores in genetic knowledge compared with other specialties. Furthermore, higher mean were reported in students who did not have a family history of genetic diseases compared with those who did. The students whose parents did not have consanguineous relativity showed significantly higher genetic knowledge than those who reported any degree of parental consanguinity. Students who had learned genetic courses had a significantly higher mean of genetic knowledge. Finally, those who were residents of cities achieved greater genetic knowledge scores. Results are shown in Table 4.

**Table 4:** Genetic familiarity variation based on sample characteristics among all participants (n = 624)

Variable		n	Genetic familiarity			Genetic knowledge		
			Mean	SD	p-value	Mean	SD	p-value
Age groups	Less than 21 years	367	4.72	1.41	0.021	12.26	2.89	< 0.001
	21 years or more	257	5.00	1.60		10.39	4.24	
Gender	Male	253	4.34	1.52	< 0.001	10.25	4.10	< 0.001
	Female	371	5.18	1.38		12.34	2.99	
Year of Study	First	153	4.13	1.16	< 0.001	12.29	2.26	< 0.001
	Second	172	5.29	1.36		12.80	2.87	
	Third	84	4.30	1.52		8.95	4.22	
	Fourth	116	5.03	1.66		10.84	3.76	
	Fifth	57	4.97	1.55		10.25	4.18	
	Sixth	42	5.95	0.94		11.76	4.54	
Family history of genetic disease	No	382	4.96	1.49	0.013	12.24	3.02	< 0.001
	Yes	242	4.65	1.48		10.31	4.17	
Parents consanguinity	No	399	5.05	1.47	< 0.001	12.49	2.77	< 0.001
	Yes, the first cousin	128	4.34	1.48		10.01	3.85	
	Yes, double the first cousin	44	4.20	1.25		6.95	4.11	
	Yes, more distant	53	4.95	1.53		11.30	4.25	
Learning of genetic courses	No	323	4.11	1.35	< 0.001	10.44	3.62	< 0.001
	Yes	301	5.62	1.23		12.61	3.29	
Place of residence	City	256	5.15	1.46	< 0.001	12.53	2.73	< 0.001
	Village	303	4.81	1.46		11.46	3.75	
	Camp	65	3.72	1.24		7.52	3.43	
Field of study	Medicine, Basic phase	120	5.27	1.26	< 0.001	13.18	2.67	< 0.001
	Medicine, clinical phase	88	6.01	0.92		13.01	2.74	
	Health Sciences	209	4.95	1.32		12.64	2.24	
	Scientific, nonmedical	102	4.36	1.73		10.79	3.43	
	Literary	105	3.59	1.13		6.67	3.32	

### Variation in genetic familiarity and genetic knowledge based on sample characteristics among medical student participants

The variation in genetic familiarity and knowledge scores among medical students based on their characteristics is shown in Table 5. Genetic familiarity was significantly variable based on age group, year of study, and learning genetic courses (p-value < 0.05). The mean was higher in students aged 21 years, students in their sixth year of study, and students who had learned genetic courses. Regarding genetic knowledge, scores remained significantly variable based on the year of study, family history of genetic disease, parental consanguinity, and learning genetic courses (p-value < 0.05). The mean was higher among sixth-year students, those who do not have a family history of genetic disease and no parental consanguinity, and students who had learned genetic courses.

**Table 5:** Variation in genetic familiarity and genetic knowledge variation based on sample characteristics among medical student participants (n = 417), significant P-values are shown in bold

Variable		Genetic familiarity				Genetic knowledge			
		n	Mean	SD	p-value	n	Mean	SD	p-value
Age groups	Less than 21 years	288	4.98	1.30	**< 0.001**	288	12.99	2.11	0.174
	21 years or more	129	5.91	1.01		129	12.63	3.16	
Gender	Male	137	5.18	1.31	0.357	137	12.69	3.04	0.277
	Female	280	5.31	1.28		280	12.97	2.16	
Year of Study	First	128	4.25	1.05	**< 0.001**	128	12.65	1.96	**< 0.001**
	Second	149	5.56	1.16		149	13.44	1.88	
	Third	26	5.38	1.46		26	11.38	4.16	
	Fourth	53	5.90	1.02		53	12.04	3.39	
	Fifth	29	6.02	0.81		29	13.00	2.46	
	Sixth	32	6.20	0.76		32	13.66	2.51	
Family history of genetic disease	No	281	5.22	1.34	0.299	281	13.09	2.19	**0.013**
	Yes	136	5.36	1.17		136	12.44	2.97	
Parents consanguinity	No	306	5.29	1.32	0.269	306	13.17	1.98	**< 0.001**
	Yes, the first cousin	60	5.30	1.19		60	12.22	3.03	
	Yes, double the first cousin	16	4.64	1.02		16	9.31	4.80	
	Yes, more distant	35	5.31	1.27		35	13.06	2.62	
Learning of genetic courses	No	184	4.68	1.25	**< 0.001**	184	12.42	2.39	**0.001**
	Yes	233	5.73	1.12		233	13.24	2.50	
Place of residence	City	198	5.29	1.37	0.951	198	13.01	2.15	0.129
	Village	205	5.25	1.23		205	12.83	2.71	
	Camp	14	5.27	1.07		14	11.64	3.20	

### Variation in genetic familiarity and genetic knowledge based on sample characteristics among non-medical student participants

The variation in the genetic familiarity and knowledge scores among non-medical students based on their characteristics is shown in Table 6. Genetic familiarity was a significant variable based on all studied variables except the age group (p-value < 0.05) while genetic knowledge was significantly variable based on all studied variables (p-value < 0.05). Females, students with no family history of genetic disease, students who reported no parental consanguinity, and who had learned genetic courses achieved higher scores in both genetic familiarity and knowledge. Based on age, the younger students had higher genetic knowledge. Sixth-year non-medical students achieved significantly higher familiarity scores, however, first-year students had significantly higher knowledge scores. Results are shown in Table 6.

**Table 6:** Variation in genetic familiarity and genetic knowledge variation based on sample characteristics among non-medical student participants (n = 207), significant P-values are shown in bold

Variable		n	Genetic familiarity			Genetic knowledge		
			Mean	SD	p-value	Mean	SD	p-value
Age groups	Less than 21 years	79	3.78	1.39	0.155	9.61	3.72	**0.009**
	21 years or more	128	4.09	1.56		8.14	4.00	
Gender	Male	116	3.34	1.08	**< 0.001**	7.37	3.24	**< 0.001**
	Female	91	4.77	1.59		10.40	4.14	
Year of Study	First	25	3.53	1.46	**0.015**	10.48	2.83	**< 0.001**
	Second	23	3.54	1.27		8.70	4.50	
	Third	58	3.81	1.29		7.86	3.79	
	Fourth	63	4.30	1.75		9.83	3.79	
	Fifth	28	3.89	1.38		7.39	3.67	
	Sixth	10	5.14	1.05		5.70	4.32	
Family history of genetic disease	No	101	4.21	1.63	**0.023**	9.87	3.69	**< 0.001**
	Yes	106	3.74	1.33		7.58	3.89	
Parents consanguinity	No	93	4.28	1.64	**0.01**	10.26	3.70	**< 0.001**
	Yes, the first cousin	68	3.49	1.16		8.06	3.44	
	Yes, double the first cousin	28	3.95	1.32		5.61	3.00	
	Yes, more distant	18	4.24	1.77		7.89	4.78	
Learning of genetic courses	No	139	3.35	1.07	**< 0.001**	7.83	3.30	**< 0.001**
	Yes	68	5.23	1.47		10.49	4.56	
Place of residence	City	58	4.67	1.64	**< 0.001**	10.90	3.73	**< 0.001**
	Village	98	3.91	1.50		8.60	4.00	
	Camp	51	3.30	0.91		6.39	2.51	

To control potential confounding factors, the variables that were significantly associated with the t-tests or in the ANOVA were included in multiple linear regression models. The models showed that higher familiarity scores of the medical students could be predicted by year of study and learning of genetic courses (Table 7). Higher knowledge scores of the medical students could be predicted by family history of genetic disease, parents’ consanguinity, and learning of genetic courses.

On the other hand, higher familiarity scores of the non-medical students could be predicted by gender, year of study, learning of genetic courses, and place of residence (Table 7). Higher knowledge scores of the non-medical students could be predicted by parents’ consanguinity, learning of genetic courses, and place of residence.

**Table 7:** Predictors of higher familiarity and knowledge scores of the medical and non-medical students

Field	Variable	Unstandardized Coefficients	SE	Standardized Coefficients	t	p-value
**Medical students**	**Familiarity**					
	Age	-0.07	0.06	-0.12	-1.35	0.178
	Year of Study	0.36	0.07	0.44	4.91	< 0.001
	Learning of genetic courses	0.61	0.13	0.23	4.75	< 0.001
	**Knowledge**					
	Year of Study	-0.07	0.09	-0.04	-0.74	0.460
	Family history of genetic disease	-0.51	0.25	-0.10	-2.00	0.047
	Parents consanguinity	-1.01	0.28	-0.18	-3.68	< 0.001
	Learning of genetic courses	0.98	0.27	0.20	3.60	< 0.001
**Non-medical students**	**Familiarity**					
	Gender	0.90	0.18	0.30	4.87	< 0.001
	Year of Study	0.28	0.06	0.25	4.48	< 0.001
	Family history of genetic disease	-0.12	0.17	-0.04	-0.73	0.464
	Parents consanguinity	-0.12	0.17	-0.04	-0.70	0.484
	Learning of genetic courses	1.21	0.19	0.38	6.37	< 0.001
	Place of residence	-0.34	0.12	-0.16	-2.94	0.004
	**Knowledge**					
	Age	-0.28	0.58	-0.03	-0.49	0.626
	Gender	1.08	0.55	0.14	1.94	0.053
	Year of Study	-0.23	0.22	-0.08	-1.05	0.293
	Family history of genetic disease	-0.80	0.50	-0.10	-1.60	0.111
	Parents consanguinity	-1.55	0.51	-0.19	-3.01	0.003
	Learning of genetic courses	1.62	0.57	0.19	2.83	0.005
	Place of residence	-1.31	0.35	-0.24	-3.78	< 0.001

## Discussion

Genetic knowledge and familiarity with genetic terms and genetic knowledge had not been previously assessed among the Palestinian population to the best of our knowledge. Our study is the leading one that assessed genetic these concepts among Palestinian university students. University students were targeted because they play central roles in raising genetic awareness due to their sway over the general population. In addition, the ages of university students impose on them to make informed decisions, especially in marriage issues that are related to genetics.

In the current study, the mean genetic familiarity and the total genetic knowledge scores among Palestinian university students were slightly lower than the 70% cut value of the total scores. Participants showed variable familiarity with the studied genetic terms. It is obvious that the majority of participants were familiar with general terms like genetics and chromosome but were less familiar with advanced and specialized terms such as vulnerability and sporadic. Variations in genetic familiarity between different terms were previously reported [12, 18]. The same conclusion applies to genetic knowledge where participants correctly answered many basic questions about gene structure and genetic diseases, but were unable to answer more advanced and specific questions in the field. Our study results are thus in line with the results of previous studies done on undergraduate students in Ecuador and the United States [9, 19]. Good knowledge about genetic diseases and genetic testing among Malaysian Medical students and good familiarity among Indonesian medical students were previously reported [20]. In contrast, poor knowledge of genetic tests among medical students and physicians in Cameroon and insufficient knowledge about genetic diseases among physicians in the Netherlands were reported [21, 22].

This leads to the conclusion that Palestinian university students lack sufficient advanced knowledge in genetics even though they showed satisfactory knowledge of general genetic information. These results were expected since general terms in genetics are taught to students at the school level. Moreover, these general terms are commonly mentioned in social media, television, and newspapers. On the other hand, more advanced and specific genetic information is expected to be explained in specialized genetic courses. Upon consulting the websites of the respective universities, it was observed that the curricula predominantly feature a minimal two-credit-hour foundational course solely mandated for specific programs such as Doctor of Medicine, Doctor of Laboratory Sciences, Dentistry, and Biotechnology. In some other health-related programs, a basic genetic course is offered as an elective, while the remaining university programs do not incorporate any coursework in genetics. These findings are alarming because adopting proper decisions about important genetic issues should be based on advanced rather than general information in medical genetics. This issue is vital in Palestine due to the increased incidence of various genetic disorders [4].

The genetic familiarity score among university students was lower than the score reported by a recent study from Indonesia [18]. However, it should be noted that our study was performed on students from different specialties while the Indonesian one was performed on medical students. Indeed, genetic familiarity among Palestinian medical students was significantly higher than that among non-medical students. Nevertheless, the genetic familiarity scores were comparable among both Palestinian and Indonesian medical students [18]. The current study showed a lower genetic familiarity score and a higher genetic knowledge score in comparison with what has been reported in a previous study from the United States [12]. This could be explained by the nature of the study samples as it was university students in our sample while it was the general adult population in the United States. It is anticipated that university students have greater knowledge than the general population. However, the wide availability of genetic counseling and services led to higher genetic familiarity among the United States population [12].

The present study concluded that the main significant predictors of genetic knowledge among university students were the field of study, age, gender, and academic year. These results are consistent with a study on university students in Saudi Arabia [23]. It is noteworthy that higher scores of genetic familiarity were found in our study among the medical students in the clinical phase compared with students in the basic phase and accordingly among sixth-year students. In contrast, higher genetic knowledge was reported among first-year students than among non-medical students separately and this could be explained by the fact that the scientific stream at the Palestinian schools usually introduces genetic facts and several molecular biology concepts. This is in agreement with the Indonesian study, and it could be attributed to the observation of clinical cases in hospitals by clinical medical students. Females had higher genetic familiarity scores in our study similar to what has been reported in a previous Australian study [24] and in contrast to what has been reported in the Indonesian study [18]. Students who are living in camps showed a significantly lower level of genetic knowledge. This could be attributed to the impact of socio-economic factors that were also evidenced among the Australian general population which found that higher income was associated with better genetic knowledge among participants [25]. In a previous study among Finns, genetic knowledge was not significantly related to gender, but younger people and those with a higher level of education exhibited greater knowledge [17]. The present study found that being female, having a higher level of education, and seeking genetic information from other people or the internet were significantly associated with higher genetic knowledge. These findings are consistent with previous studies [26, 27]. Previous studies found that females had better health-promoting lifestyles, health responsibility, and health knowledge than males [28, 29]. The interest of females in genetic knowledge more than males could be expected because genetic disorders and reproduction issues are a primary concern of females as current or future mothers. In addition, it was reported that females tend to accept medical information objectively while males tend to reject medical information if it conflicts with the traditional culture [30]. However, gender was not a significant factor in either genetic familiarity or knowledge among medical students separately.

The field of study was a significant predictor in determining genetic knowledge and familiarity among students. Indeed, medical and health sciences students scored higher than non-medical students in genetics familiarity and knowledge. This is expected as genetic courses are usually part of their university curriculum besides being previously introduced to some genetic terms at the high school level. Notably, second-year medical students achieved significantly higher genetic knowledge scores. This could be attributed to the fact that medical students should attend the genetics course during their second year of study according to the adopted medical curriculum in Palestine. The circular integration of genetic knowledge in academic curriculums based on extensive studies is important [31].

The current findings reported that consanguineous marriages are still a rooted social and cultural behavior among Palestinians as more than one-third of the sample that took part in this study reported consanguineous marriage in their families [32]. Although this prevalence of consanguinity is less than the latest report from the Palestinian Central Bureau of Statistics for the year 2021, it is worrying because the prevalence of consanguinity is still high even in educated families due to the Arab cultural background [33]. Low genetic knowledge was shown to be associated with a higher consanguinity rate in previous studies [33]. University students were the focus of this study as they represent the prospective community stakeholders. If well prepared, they can play positive roles in debating the awareness in the population. They also fall within the age of pre-marriage. The data gathered from this study may contribute to the importance of improving the contents of the current genetic curriculum and the development of programs to reinforce the teaching of genetics. In order to address the pressing need for enhanced genetic knowledge in Palestine, interventions should not only be implemented at the university level, irrespective of students’ academic majors but also commence with the reinforcement of genetic education within schools. This is imperative due to the high prevalence of genetic disorders and the prevalent practice of consanguineous marriages in the region. Non-pedagogical initiatives, such as organizing seminars and awareness campaigns overseen by genetics professionals, can be undertaken by well-educated and trained university students. These endeavors will serve to increase public awareness and mitigate the stigma associated with genetic diseases. Ultimately, these interventions will yield positive national-level outcomes by fostering a comprehensive understanding of genetic concepts and facilitating informed decision-making, particularly in matters pertaining to marriage and genetic testing. Notably, two published studies conducted in neighboring Arab countries (Jordan and Saudi Arabia) have demonstrated a positive correlation between university education and favorable attitudes towards genetic testing [23, 34]. Enhancing genetics education will also facilitate research efforts pertaining to genetic diseases in Palestine.

### Strengths and limitations of the study

This is the first study that assessed the important concepts of genetic familiarity and genetic knowledge using a relatively large sample from ten universities with different fields of study in Palestine. Previously validated scales that were applied by several international studies were used in the present study. However, there were some limitations such as the subjective scoring of genetic familiarity. Furthermore, it was difficult to determine the response rate of students as the questionnaire was distributed online. Answering the questionnaire was self-reported so we can not exclude the recall bias in participants’ answers.

## Conclusions

The current study demonstrated that genetic knowledge and familiarity among Palestinian university students is slightly low, especially in advanced and specific genetic information. Significant variations in genetic knowledge and familiarity among the students were observed depending on the field of study, year of study, gender, place of residence, parental consanguinity, and family history of genetic diseases. Consanguinity, as a well-known risk factor for genetic disorders, is still deeply rooted and practiced in Palestine as it was shown to be the case in over a third of the sample that took part in this study.

## Data Availability

All data used in the study are available upon request from the corresponding author.
